# Cytoreductive surgery offers prognostic benefits in metastatic gastrointestinal stromal tumors with generalized progression following imatinib therapy: a single institute retrospective study

**DOI:** 10.1186/s12893-023-02087-3

**Published:** 2023-07-04

**Authors:** Dao-Ning Liu, Wei-Wei Jia, Hai-Yue Wang, Jian-Hui Wu, Cheng-Peng Li, Chun-Yi Hao

**Affiliations:** 1 Key Laboratory of Carcinogenesis and Translational Research (Ministry of Education/Beijing), Department of Hepato-Pancreato-Biliary Surgery/Sarcoma Center, Peking University Cancer Hospital & Institute, No. 52 Fucheng Road, Haidian District, Beijing, People’s Republic of China; 2 Key Laboratory of Carcinogenesis and Translational Research (Ministry of Education/Beijing), Department of Pathology, Peking University Cancer Hospital & Institute, Beijing, People’s Republic of China

**Keywords:** Gastrointestinal stromal tumors, Cytoreductive surgery, Prognosis, Tyrosine kinase inhibitor, Surgical strategy

## Abstract

**Background:**

Gastrointestinal stromal tumors (GISTs) are the most common mesenchymal tumors of the gastrointestinal tract. Distant metastasis has been detected in approximately 50% of GIST patients at the first diagnosis. The surgical strategy for metastatic GIST with generalized progression (GP) after imatinib therapy remains unclear.

**Methods:**

We recruited 15 patients with imatinib-resistant metastatic GIST. They received cytoreductive surgery (CRS) for tumor rupture, intestinal obstruction and gastrointestinal bleeding. We collected clinical, pathological and prognostic data for analyses.

**Results:**

OS and PFS after R0/1 CRS were 56.88 ± 3.47 and 26.7 ± 4.12 months, respectively, when compared with 26 ± 5.35 and 5 ± 2.78 months after R2 CRS (*P* = 0.002 and *P* < 0.001, respectively). The OS of patients from the initiation of imatinib in the R0/1 group was 133.90 ± 15.40 months when compared with 59.80 ± 10.98 months in the R2 CRS group. There were two significant grade III complications after 15 operations (13.3%). No patient underwent reoperation. In addition, no perioperative death occurred.

**Conclusions:**

R0/1 CRS is highly probable to provide prognostic benefits for patients with metastatic GIST who experience GP following imatinib treatment. An aggressive surgical strategy for achieving R0/1 CRS can be deemed safe. If applicable, R0/1 CRS should be carefully considered in imatinib-treated patients with GP metastatic GIST.

## Introduction

Gastrointestinal stromal tumors (GISTs) are the most common mesenchymal tumors of the gastrointestinal tract. GISTs arises from the visceral interstitial cells of the Cajal [1]. Distant metastasis has been detected in approximately 50% of GIST patients at the first diagnosis [2]. Tyrosine kinase inhibitors (TKIs) and surgery are primary therapies for metastatic GISTs. Complete resection for metastatic GIST is beneficial in patients receiving imatinib or sunitinib who exhibit a radiographic response or limited disease progression (LP) [3]. However, the surgical strategy for metastatic GIST with generalized progression (GP) after imatinib therapy remains unclear.

Cytoreductive surgery (CRS), the main surgical strategy for metastatic GIST, comprises a series of resections dependent on disease dissemination [2, 4, 5]. Considering imatinib-treated patients with metastatic GIST, the progression-free survival (PFS) after CRS was 11 and 6 months for LP and GP, respectively, whereas the overall survival (OS) after CRS was 59 and 24 months for LP and GP, respectively [2]. Compared with the PFS and OS associated with sunitinib for imatinib-resistant metastatic GIST (34 weeks and 107 weeks, respectively) [1], the PFS and OS post-metastasectomy in patients with metastatic GIST who experienced GP after imatinib therapy (6 months and 24 months, respectively) seemed to be even shorter.

However, based on our clinical experience, combining imatinib, sunitinib, regorafenib, and other TKI therapies after CRS could considerably increase the OS and PFS in imatinib-treated patients with metastatic GIST experiencing GP when compared with those previously reported [2, 6]. Although several studies have examined the potential of CRS for metastatic GIST, TKI therapy post-CRS remains poorly explored [7]. On the other hand, a high percentage of R2 resection in imatinib-treated patients with GP may explain the short OS and PFS reported in former studies [2, 7]. Herein, we enrolled patients with metastatic GIST who received standardized TKI therapy after CRS to determine whether CRS can offer prognostic benefits to patients with metastatic GIST exhibiting GP after imatinib treatment.

## Materials and methods

### Patients

Herein, we recruited 15 patients with imatinib-resistant GIST. All patients underwent CRS at the Peking University Cancer Hospital Sarcoma Center during an 8-year period (March 2013 to August 2020). Ethical approval and written informed consent were obtained from all participants. We collected clinical, pathological, and prognostic information. The prognostic groups and pathological characteristics of GISTs were reassessed by two experienced pathologists blinded to clinical and prognostic data [8].

### Inclusion criteria for prognostic analysis

1. Patients with preoperative diagnosis and postoperative pathology of GIST were included.
2. Patients who received imatinib before surgery and response evaluation were GP were included. GP was defined as a multifocal progressive disease.
3. All patients signed the informed consent forms and agreed to participate in the study.
4. Patients with distant metastases were included. No metastasis was observed outside of the abdomen in this cohort.

### Exclusion criteria for prognostic analysis

(1) Patients with non-tumor-related death were excluded (one patient who died of a heart attack two years after CRS was excluded).

### TKI therapy

In patients with GISTs, we employed imatinib as first-line therapy and sunitinib as second-line therapy. Additional therapies administered after imatinib and sunitinib failure are listed in Tables 1 and 2, respectively. The mutational status of primary and resistant metastatic tumors are listed in Table 2. Patients generally resumed the same TKI therapy upon discharge or after their first postoperative visit. Imatinib dose escalation (400 to 600 mg) was the first choice after CRS. For patients with secondary mutations or severe adverse events after imatinib therapy, sunitinib was recommended by a multidisciplinary team [9]. This cohort was selected from patients treated over 8 years. Accordingly, some TKI therapies might not be in accordance with current guidelines, given that the employed guidelines were based on the perception at the time of treatment. Five patients did not receive regorafenib as third-line therapy or ripretinib as fourth-line therapy.

**Table 1:** Clinical and pathological characters for patients with metastatic GIST who received R0/1 or R2 CRS

**Parameters**	**n**	**R0/1 Resection**	**R2 Resection**	***P*-value**
Age (y)		52.90 ± 8.556	57.20 ± 4.970	0.164
Operative time (min)		326.40 ± 115.64	350.40 ± 120.34	0.902
Blood loss (ml)		1210.00 ± 1567.34	700.00 ± 538.52	0.180
Gender				0.680
Male	11 (73.4%)	7 (46.7%)	4 (26.7%)	
Female	4 (26.7%)	3 (31.9%)	1 (6.7%)	
Tumor size (cm)		13.77 ± 8.67	14.80 ± 7.29	0.489
Primary tumor site				< 0.001
Gastric GIST	3 (20%)	2 (13.3%)	1 (6.7%)	
Non-gastric GIST	12 (80%)	8 (53.3%)	4 (26.7%)	
Reason of surgery				0.392
Tumor rupture	8 (53.3%)	4 (26.7%)	4 (26.7%)	
Intestinal obstruction	5 (33.3%)	4 (26.7%)	1 (6.7%)	
Gastrointestinal bleeding	2 (13.3%)	2 (13.3%)	0 (0%)	
Complication (Dindo–Clavien Classification)				0.567
I	9 (60%)	7 (46.7%)	2 (13.3%)	
II	4 (26.7%)	3 (20%)	1 (6.7%)	
IIIa	1 (6.7%)	0 (0%)	1 (6.7%)	
IIIb	1 (6.7%)	1 (6.7%)	0 (0%)	
Length of stay (days)		24.80 ± 9.34	25.60 ± 10.41	0.836
Resection of organs		2.70 ± 1.58	3.40 ± 1.34	0.898
Organs invaded by GIST		1.00 ± 0.47	1.60 ± 0.89	0.038
Genetic mutation				0.531
KIT exon 11	5 (33.3%)	3 (20%)	2 (13.3%)	
KIT exon 9	3 (20%)	3 (20%)	0 (0%)	
KIT exon 11 + 17	2 (13.3%)	1 (6.7%)	1 (6.7%)	
Wild type	2 (13.3%)	1 (6.7%)	1 (6.7%)	
KIT exon 17	1 (6.7%)	1 (6.7%)	0 (0%)	
KIT exon 11 + 13	1 (6.7%)	0 (0%)	1 (6.7%)	
KIT exon 11 + PDGFRA	1 (6.7%)	1 (6.7%)	0 (0%)	
Location of metastases				0.153
liver only	2 (13.3%)	1 (6.7%)	1 (6.7%)	
peritoneum only	5 (33.3%)	5 (33.3%)	0 (0%)	
both liver and peritoneum	8 (53.3%)	4 (26.7%)	4 (26.7%)	
TKI therapy after CRS				0.497
Sunitinib	4 (26.7%)	3 (20%)	1 (6.7%)	
Imatinib + Sunitinib	3 (20%)	2 (13.3%)	1 (6.7%)	
Imatinib	2 (13.3%)	2 (13.3%)	0 (0%)	
Imatinib + Sunitinib + Dasatinib	2 (13.3%)	1 (6.7%)	1 (6.7%)	
Imatinib + Sunitinib + Regorafenib	1 (6.7%)	0 (0%)	1 (6.7%)	
Imatinib + Sunitinib + Ripretinib	1 (6.7%)	1 (6.7%)	0 (0%)	
Sunitinib + Regorafenib + Dasatinib	1 (6.7%)	0 (0%)	1 (6.7%)	
Imatinib + Dasatinib + Regorafenib	1 (6.7%)	1 (6.7%)	0 (0%)	
T Stage				0.662
T1	1 (6.7%)	1 (6.7%)	0 (0%)	
T2	0 (0%)	0 (0%)	0 (0%)	
T3	4 (26.7%)	3 (20%)	1 (6.7%)	
T4	10 (66.7%)	6 (40%)	4 (26.7%)	
N Stage				
N0	15 (100%)	10 (66.7%)	5 (33.3%)	
N1	0 (0%)	0 (0%)	0 (0%)	
Mitotic Rate				1.000
Low (≤ 5/50HPF)	6 (40%)	4 (26.7%)	2 (13.3%)	
High (> 5/50HPF)	9 (60%)	6 (40%)	3 (20%)	
Prognostic Category				0.654
1	1 (6.7%)	1 (6.7%)	0 (0%)	
3b	1 (6.7%)	1 (6.7%)	0 (0%)	
6a	4 (26.7%)	3 (20%)	1 (6.7%)	
6b	9 (60%)	5 (33.3%)	4 (26.7%)	
Number of metastases				0.171
≤ 5	3 (20%)	3 (20%)	0 (0%)	
> 5	12 (80%)	7 (46.7%)	5 (33.3%)

**Table 2:** TKI therapies and tumor mutational status of the cohort

	Mutational status of primary tumors	Mutational status of resistant metastatic tumors	Death or not at the last follow-up	TKI therapy after CRS
Case 1	KIT exon 9	KIT exon 9	No	Imatinib
Case 2	KIT exon 11	KIT exon 11	Yes	Imatinib + Sunitinib + Regorafenib
Case 3	KIT exon 9	KIT exon 9	Yes	Sunitinib
Case 4	KIT exon 11	KIT exon 11 + 17	Yes	Sunitinib + Regorafenib + Dasatinib
Case 5	KIT exon 9	KIT exon 9	No	Imatinib + Sunitinib
Case 6	KIT exon 11	KIT exon 11 + 13	No	Sunitinib
Case 7	KIT exon 11	KIT exon 11	Yes	Imatinib + Sunitinib
Case 8	KIT exon 11	KIT exon 11	No	Imatinib
Case 9	KIT exon 11	KIT exon 11 + 17	No	Sunitinib
Case 10	KIT exon 11	KIT exon 11	Yes	Imatinib + Sunitinib
Case 11	KIT exon 11	KIT exon 11	No	Imatinib + Sunitinib + Ripretinib
Case 12	KIT exon 11 + PDGFRA	KIT exon 11 + PDGFRA	No	Sunitinib
Case 13	KIT exon 17	KIT exon 17	No	Imatinib + Dasatinib + Regorafenib
Case 14	Wild type	Wild type	Yes	Imatinib + Sunitinib + Dasatinib
Case 15	Wild type	Wild type	Yes	Imatinib + Sunitinib + Dasatinib

We performed abdominal and pelvic computed tomography with contrast medium every three months after the primary diagnosis and first-line TKI therapy for follow-up. Based on the analysis of serial preoperative imaging using either RECIST or Choi criteria, the radiographic response at the time of surgery was categorized as responsive disease, stable disease, limited progressive disease (progression at a single site of disease with all other sites responsive or stable), and generalized progressive disease (progression at more than one site).

### Cytoreductive surgery

**Fig. 1:**
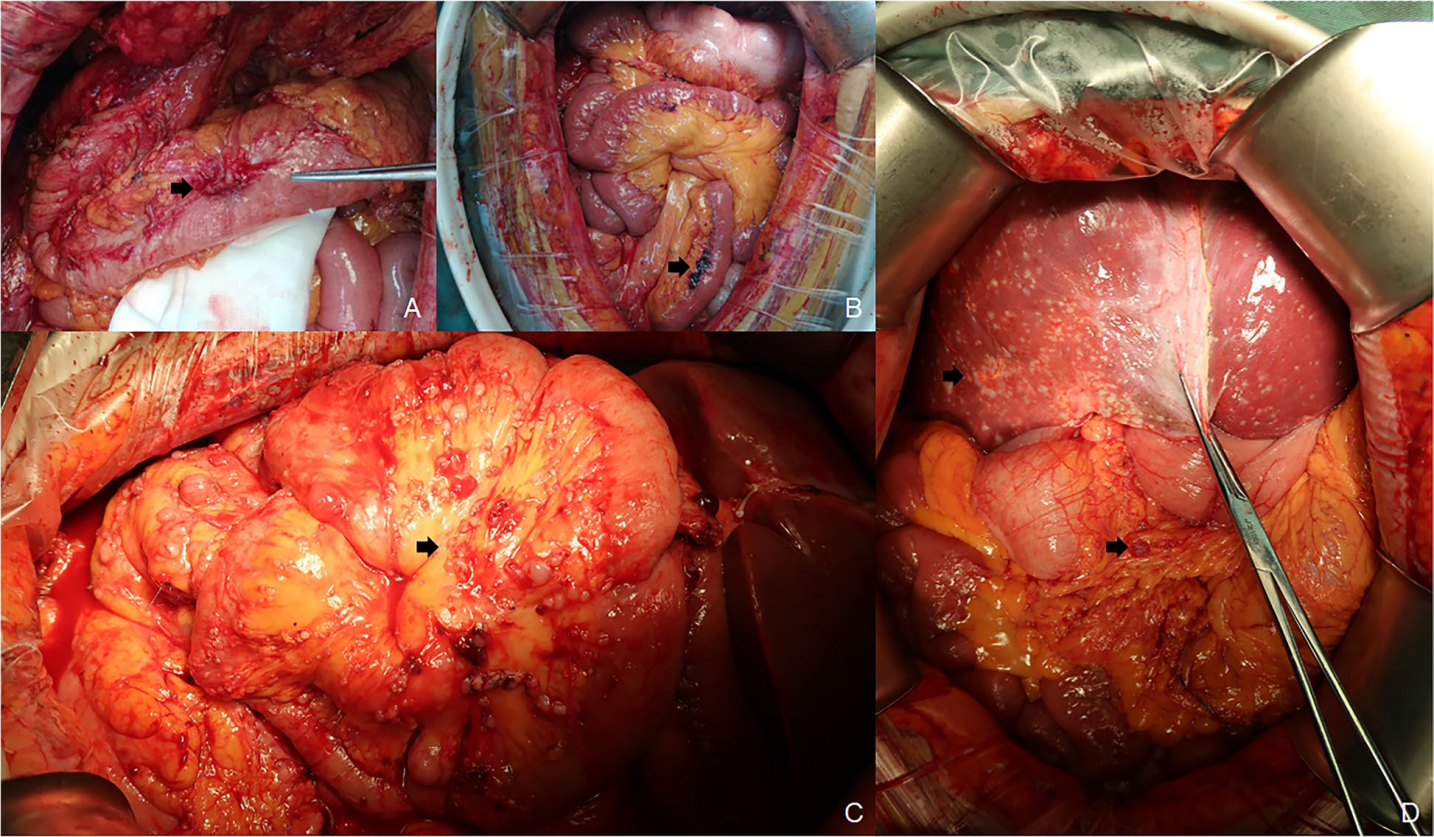
**A** and **B** show mesenteric metastases and R0/1 CRS. **C** and **D** show metastatic GIST with wide dissemination. Only R2 resection could be performed for these patients

Imatinib was discontinued after the radiographic response was preoperatively categorized as GP. The goal of resection was to excise all visible disease in patients. The completeness of resection with CRS was assessed by the surgeon at the end of the procedure and classified into two categories: R0/1 signified no macroscopic residual cancer and R2 signified macroscopic residual cancer (Fig. 1). Postoperative complications were categorized using the Clavien-Dindo classification system [10]. The location of metastases was defined as the liver only, peritoneum only, and both the liver and peritoneum.

### Pathology

Resected primary tumors and metastases were subjected to mutation analysis. Testing was performed for mutation sites of KIT (exons 9, 11, 13, 14, and 17), PDGFRA (exons 12, 18, and D842 V), SDH, BRAF, and NF1. The metastatic mitotic index was defined as the number of mitoses per 50 high-power fields (HPF) in resected metastatic specimens.

### Statistics

Data collection and statistical analyses were performed using IBM SPSS Version 26 (SPSS Inc., Chicago, IL, USA). Numerical data are expressed as mean and standard deviation, ranked data by cross-tabulation and percentages. PFS and OS were estimated by the Kaplan–Meier method. PFS was calculated from date of CRS. OS was calculated from both date of CRS and date of TKI initiation. The log-rank test was used to determine statistical differences in OS and PFS. Univariate regression analysis was performed using a Cox proportional hazards model. Statistical analyses were performed using the t-test, linear regression, analysis of variance (ANOVA), nonparametric tests, chi-square test, and log-rank test. All tests were two-sided, with a significance level of *P* = 0.05.

## Results

Herein, the median tumor size was 14 cm (range: 1–28 cm). The most common primary tumor sites were the small bowel (*n* = 11, 73.3%), stomach (*n* = 3, 20%), and colon (*n* = 1, 6.7%). Metastases were identified in the peritoneum alone (*n* = 5, 33.3%), the liver alone (*n* = 2, 13.3%), or simultaneously in the peritoneum and liver (*n* = 8, 53.3%). Resections were macroscopically complete (R0/R1) in 10 patients (66.7%) and incomplete (R2) in 5 patients (33.3%). The median duration of preoperative imatinib therapy was 31 months (range: 6–145 months). The median follow-up period for the entire cohort was 38 months (range: 12–63 months) from the time of CRS and 81 months (range, 26–167 months) from the initiation of imatinib therapy.

**Fig. 2:**
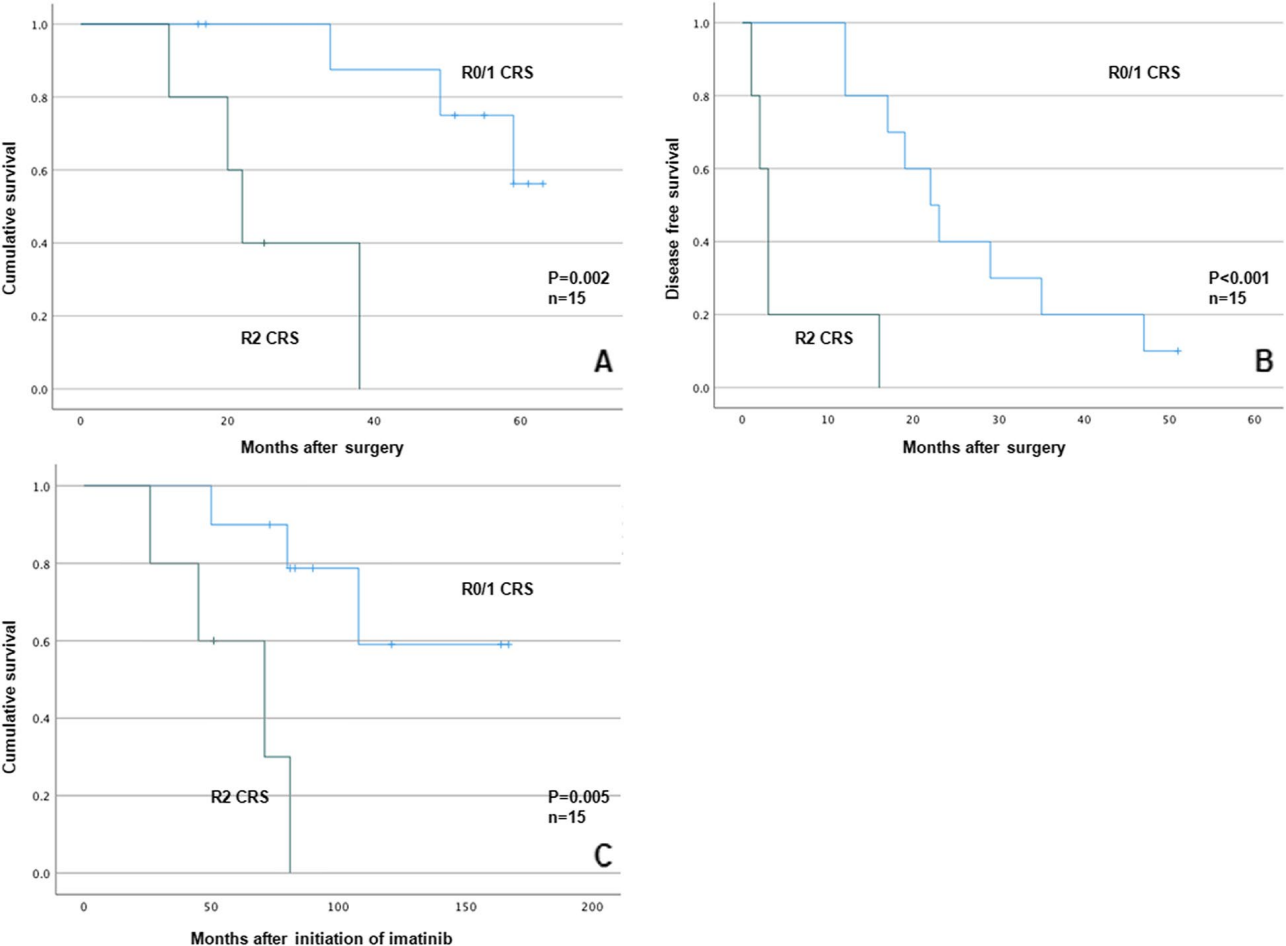
**A** and **B** show PFS and OS from the date of CRS by the extent of resection (R0/1 or R2). **C** shows OS from the date of imatinib initiation by the extent of resection

OS and PFS after R0/1 CRS were 56.88 ± 3.47 and 26.7 ± 4.12 months, respectively, when compared with 26 ± 5.35 and 5 ± 2.78 months after R2 CRS (*P* = 0.002 and *P* < 0.001, respectively, Fig. 2). The clinical and pathological characteristics of the patients are listed in Table 1. The OS of patients from the initiation of imatinib in the R0/1 group was 133.90 ± 15.40 months when compared with 59.80 ± 10.98 months in the R2 CRS group (*P* = 0.005, Fig. 2). The PFS of imatinib before CRS was 46.60 ± 39.06 months.

Univariate analysis revealed that the duration of preoperative imatinib use (≤ 24 or > 24 months), location of metastasis (liver only, peritoneum only, both liver and peritoneum), metastatic mitotic index (≤ 5/50 or > 5/50 HPF), and the number of metastases (≤ 5 or > 5) failed to predict PFS and OS (*P* = 0.146, 0.537, 0.164, 0.430, 0.997, 0.061, 0.514, and 0.611, respectively, Table 3). We did not perform a multivariate analysis owing to the small sample size.

**Table 3:** Univariate analysis of treatment and tumor variables on progression-free survival and overall survival for all patients with metastatic GIST experiencing generalized progression after imatinib treatment at time of cytoreductive surgery

Variables	Progression-free survival		Overall survival	
	Hazard Ratio (CI)	*P*-value	Hazard Ratio (CI)	*P*-value
Duration of preoperative TKI		0.146		0.997
≤ 24 mo	Ref		Ref	
> 24 mo	0.429 (0.13–1.41)		0.99 (0.22–4.56)	
Location of disease		0.537		0.061
Liver only	Ref		Ref	
Peritoneum only	2.49 (0.45–13.68)		1.83 (0.33–10.08)	
Both	1.15 (0.35–3.78)		1.28 (0.96–3.25)	
Metastatic mitotic index		0.164		0.514
< 5/50 HPF	Ref		Ref	
≥ 5/50 HPF	2.4 (0.66–8.78)		1.64 (0.37–7.35)	
Number of metastases		0.430		0.611
≤ 5	Ref		Ref	
> 5	1.72 (0.43–6.92)		0.581 (0.07–4.85)	
Extent of resection		< 0.001		0.002
R0/1	Ref		Ref	
R2	12.55 (2.28–69.07)		16.06 (1.68–153.81)	

We resected 2.93 ± 1.49 organs and did not perform vascular resection. Fifteen patients developed six postoperative complications following 15 operations (40%). There were two significant grade III complications after 15 operations (13.3%). No patient underwent reoperation. In addition, no perioperative death occurred.

## Discussion

CRS is one of the primary strategies employed in patients with metastatic GIST. However, recent studies have suggested performing CRS only in imatinib- or sunitinib-treated patients with metastatic GIST exhibiting LP. Based on our clinical observations, imatinib-treated patients with metastatic GIST experiencing GP may also benefit from complete resection. In the present study, we enrolled patients with metastatic GIST exhibiting GP and underwent CRS for tumor rupture, intestinal obstruction, and gastrointestinal bleeding.

In patients with GIST experiencing GP, we noted that OS and PFS after R0/1 CRS were 56.88 ± 3.47 and 26.7 ± 4.12 months, respectively. These values are substantially longer than the 6-month PFS and 24-month OS recorded in imatinib-treated patients with multifocal progressive disease (MPD) after CRS, as reported by Fairweather et al. [2]. However, the results documented by Fairweather et al. are similar to those of patients who underwent R2 CRS (26 ± 5.35 and 5 ± 2.78 for OS and PFS, respectively) in the present study. In the study by Fairweather et al., the goal of resection was to remove all visible disease in patients with responsive disease and stable disease, and at minimum areas of progressive disease in patients with unifocal progressive disease (UPD) or MPD. The authors performed 125 R2 resections in 236 patients (53%) with UPD and MPD. We believe that the percentage of R2 resection should be higher in patients with MPD. Moreover, it can be suggested that the high percentage of R2 resections in imatinib-treated patients with GP may be responsible for the short OS and PFS. Hirotoshi Kikuchi's review article provided information on the complete resection rates of patients who exhibited systemic resistance after receiving imatinib in six previous studies, ranging from 0 to 39%. PFS and OS after CRS for patients with systemic resistance were reported to be 2-6 months and 5.6-26 months, respectively [7]. Only Alessandro Gronchi's study reported a complete resection rate of 100% for two patients with systemic resistance. However, the OS and PFS data for these two patients were not reported [11]. Accordingly, complete resection may be a more important factor than imatinib response evaluation. CRS should be considered for patients with metastatic GIST who experience GP following imatinib treatment if R0/1 resection is applicable.

Herein, we observed that traditional prognostic factors did not significantly impact prognosis; this finding could be attributed to our aggressive surgical strategy. We speculate that the location of metastasis, number of metastases, and metastatic mitotic index could have markedly influenced the extent of resection in previous studies. In the present study, the goal of resection was to remove all visible disease in all patients. In our study, 66.7% of patients underwent R0/1 CRS, which was considerably higher than previously reported [2].

Another drawback of recent studies assessing CRS for metastatic GIST is the lack of description regarding TKI therapy post-surgery. We believe the evaluation of CRS alone, without postoperative TKI therapy, is incomplete. With the development of TKI therapy, there is a growing number of emerging drugs and clinical trials, including ripretinib [12], dasatinib [13], larotrectinib [14], and avapritinib [15]. These novel therapeutic agents could afford prognostic benefits in patients with metastatic GIST. Therefore, the role of CRS in metastatic GIST needs to be re-evaluated considering TKI therapy post-surgery. In the present study, we supervised postoperative TKI therapy in each patient to offer an appropriate therapeutic strategy. With standardized TKI therapy, we observed that complete resection could confer prognostic benefits to patients with metastatic GIST who experience GP following imatinib treatment.

CRS can benefit imatinib-treated patients with metastatic GIST experiencing GP in several ways. First, CRS can reduce the tumor burden and offer good physical conditions for subsequent TKI therapy. Resolving issues such as severe symptoms that disrupt TKI therapy can benefit the prognosis. Secondly, mutation analysis after CRS can be valuable for discovering secondary mutations. For patients with metastatic GIST who experience GP following imatinib treatment, reanalysis of genetic mutations is vital for postoperative TKI therapy. Third, resection of rapidly growing or drug-resistant lesions may offer prognostic benefits, given that imatinib can still confer prolonged PFS in some imatinib-treated patients with metastatic GIST experiencing GP. In our cohort, ten patients with GIST received imatinib after CRS, resulting in a PFS of 16.8 ± 3.95 months. The mechanism underlying the observed prognostic benefits in imatinib-treated patients with metastatic GIST experiencing GP post-CRS warrants further elucidation.

CRS is a safe procedure for patients with GISTs. With an aggressive surgical strategy to achieve R0/1 CRS, we resected 2.93 organs and did not perform vascular resection in this cohort. No patients underwent reoperation or experienced perioperative death. Additional cases are needed to evaluate the risks of CRS in future investigations. However, unlike the study by Fairweather et al., the goal of resection was to remove the minimum areas of progressive disease in patients with GP. Based on our findings, we recommend an aggressive surgical strategy to achieve R0/1 resection.

The limitations of the present study need to be addressed. First, this study is a single institute retrospective cohort study with small sample size. To perform multivariate prognostic analysis, we need to accumulate additional cases in the future. A definitive conclusion should be drawn through a randomized clinical trial. Second, selection bias may exist in this study, since we could only perform CRS on patients with good physical conditions. Poor physical conditions caused by rapid progressing disease may lead to worse prognosis. These patients need to be taken into consideration in future study. Third, we observed that R0/1 CRS prolonged OS and PFS in imatinib-treated patients with metastatic GIST experiencing GP; however, the mechanism underlying this phenomenon remains elusive. We believe that reducing the tumor burden may improve the efficacy of TKI therapy. Lastly, patients in this cohort were recruited over an 8-year period. Therefore, some patients did not receive “standardized TKI therapy” based on a recent consensus. We believe that advances in TKI therapy in recent years can afford longer PFS and OS than those observed in this cohort. R0/1 CRS may offer prognostic benefits for sunitinib-treated patients with metastatic GIST who experience GP, and we plan to investigate this in our future research. There are several unresolved issues considering patients with metastatic GIST who experience GP following imatinib treatment, such as the timing of surgery, CRS standards, and perioperative TKI therapy. Accordingly, a study assessing CRS in patients with metastatic GIST who experience GP following imatinib treatment should be further designed.

## Conclusion

R0/1 CRS is highly probable to provide prognostic benefits for patients with metastatic GIST who experience GP following imatinib treatment. An aggressive surgical strategy for achieving R0/1 CRS can be deemed safe. If applicable, R0/1 CRS should be carefully considered in imatinib-treated patients with GP metastatic GIST.

## Data Availability

The datasets generated and analysed during the current study are not publicly available due consideration of medical ethics, but are available from the corresponding author on reasonable request.

## Abbreviations

GISTs: Gastrointestinal stromal tumors
TKIs: Tyrosine kinase inhibitors
LP: Limited disease progression
GP: Generalized progression
CRS: Cytoreductive surgery
HPF: High-power fields
MPD: Multifocal progressive disease
UPD: Unifocal progressive disease
